# Stabilisation of waterlogged archaeological wood: the analysis of structural and dimensional changes of different conservation methods using magnetic resonance imaging and X-ray micro-computed tomography

**DOI:** 10.1038/s41598-025-23385-1

**Published:** 2025-11-04

**Authors:** Jörg Stelzner, Damian Gwerder, Rolf Pohmann, Philipp Schuetz, Waldemar Muskalla, Markus Wittköpper, Katharina Schmidt-Ott, Ingrid Stelzner

**Affiliations:** 1 Leibniz-Zentrum für Archäologie, Ludwig-Lindenschmit Forum 1, 55116 Mainz, Germany; 2 Lucerne University of Applied Sciences and Arts-School of Engineering and Architecture, Technikumstrasse 21, Horw, 6048 Switzerland; 3 High-Field Magnetic Resonance, Max Planck Institute for Biological Cybernetics, Max-Planck-Ring 11, 72076 Tübingen, Germany; 4 Collection Center, Swiss National Museum, Lindenmoosstrasse 1, Affoltern am Albis, 8910 Switzerland

**Keywords:** Conservation, Waterlogged archaeological wood, Collapse, Cracks, Volume, Shrinkage, X-ray micro-computed tomography, Magnetic resonance imaging, Engineering, Materials science

## Abstract

Waterlogged archaeological wood can be preserved for many years in the absence of air, as decomposition is substantially slowed down. After excavation, conservation is necessary to prevent damage of objects due to uncontrolled drying. In this study, the following conservation methods were tested to investigate their ability to stabilise the objects: alcohol-ether resin, melamine-formaldehyde (Kauramin 800), lactitol/trehalose, saccharose, polyethylene glycol (PEG 2000 with air-drying and PEG 2000 or 400 and 4000 with subsequent freeze-drying). In order to precisely understand the changes caused by conservation and drying, 40 samples each of pine wood and oak wood were documented using magnetic resonance imaging and X-ray micro-computed tomography before and after conservation. This imaging made it possible to quantitatively record changes in the wood structure, for example due to shrinkage, collapse and cracks, which could not be prevented by conservation. The alcohol-ether-resin method with solvent drying had the best stabilizing effect and no damage of the wood structure was visible. The two PEG treatments followed by freeze drying showed effective volume stabilisation. In both cases, however, the treatment led to cracks in the wood structure, which occurred less frequently when the cryoprotectant PEG 400 was used. In comparison, the other methods with air drying did not show consistently good results in stabilizing the volume or wood structure.

## Introduction

Wood can be preserved in temperate climate zones in water bodies or water-saturated soils under anoxic conditions over a very long period of time. The degradation of wood under such conditions takes place very slowly and leads to a change in the structure and composition of the objects: Primarily, the cellulose components of the secondary cell wall are decomposed by microorganisms such as fungi (soft-rot fungi (SR)) and bacteria (tunnelling bacteria (TB) and erosion bacteria (EB))^1^. Furthermore, lignin can be degraded by oxidation and hydrolysis^2^. This degradation is a common process that leads to decay of the wood, in which ultimately only the skeletal framework of the middle lamella holds the structure together. The secondary cell wall, which is primarily responsible for the stability of the undamaged wood^3^, then contains only degraded lignin and bacterial slime^4^. The process can be very heterogeneous and some cells can be degraded while neighbouring cells remain undamaged^5^. This decomposition, which begins in the outer regions, leads to physical weakening of the structure and to fragility of the archaeological wood during ground deposition^6^. Water from the environment fills the pore spaces - the capillaries and microcapillaries - and causes the wood structure to swell. Waterlogged archaeological wood (WAW) continues to decompose, and the interaction of microorganisms, fungal attack and chemical reactions with salt and fresh water can increase this decomposition and lead to extensive damage that compromises the structural integrity of the wood remains^7^. The more cell wall material is broken down, the more water fills the internal cavities. The maximum water content therefore increases as the wood degrades and is an universally used indicator of the state of preservation^8^. WAW is very sensitive after excavation and can suffer considerable damage within a few hours without protective measures. Due to the increased oxygen supply after excavation, WAW is also susceptible to degradation by oxygen-tolerant microorganisms^1^ and other oxygen-related degradation processes. If WAW air-dries, capillary forces occur due to the high surface tension of the evaporating water. This causes the cell walls to shrink and the cells to collapse^9,10^. Depending on the degree of degradation, only a fraction of the wood volume remains. The dimensional changes during drying happen in two stages^11^: Above the fibre saturation point, degraded cells collapse due to capillary tension and drying stress^12,13^. Below the fibre saturation point, the cell walls begin to shrink, whereby the volume loss of WAW is proportional to the water content and the shrinkage in tangential direction is generally greater than in radial and longitudinal direction^10^.

Wood has unique material properties and is also readily available and easy to work with. Due to its frequent use and the information stored in wood anatomy, it plays an irreplaceable role in the study of the way of life of people and their environment in the past. This requires the permanent preservation of these unique sources, which has led to the development of methods to prevent uncontrolled drying out^14^. The damaging effect of air-drying and the question of how to solve this problem was recognized as early as the 19th century as not an easy task^15^, and since that time a variety of methods and conservation agents have been tested^14^. Currently there are two different strategies to preserve WAW: the (re-) burial in a protected, natural, anaerobic and waterlogged environment and conservation treatments. To avoid shrinkage, rupture, splits, cracks and collapse of WAW upon drying is the main challenge for the conservation^16^. In addition to the preservation of cultural heritage, conservation measures are required to minimise the impact on the material properties for possible future investigations^17^. Furthermore, other factors such as reversibility, effort, toxicity of ingredients, long-term stability, and a possible catalytic effect on the chemical degradation of the wood, such as acid hydrolysis or Fenton reactions are also of great importance when selecting a method. Currently, all methods have their positive and negative aspects. Further details are described for example here^14,18^.

In practise, conservation agents are added to the solution prior to drying. In general, they work through two main principles^19^: impregnation and/or bulking. Impregnation fills degraded wood structures (lumina, cell wall and microcapillaries) with a solidifying agent, reinforcing stability and protecting the wood during drying. Bulking fills micropores and pores of the cell walls, enhancing the wood’s resistance to drying stress and minimizing shrinkage. For a good conservation result, the conservation agents must effectively permeate, adsorb and stabilise the (residual) cell wall and/or the lumen so that collapse and shrinkage are prevented during drying^20^. For controlled drying, certain techniques such as freeze-drying or the use of solvents with low surface tension (e.g. acetone, ethanol) are used in some conservation methods to reduce capillary tension and prevent collapse^21^. In the case of freeze-drying conservation agents can also act as a cryoprotectant to prevent volume expansion of the water during freezing^22^.

Conservation methods are evaluated on how well they stabilise the object during and after drying but also in the long-term while not altering the characteristics of the wood. How successfully a method preserves the dimensions of an object can be determined by comparing the condition before and after conservation. The stabilizing quality is measured by preventing shrinkage as well as cracks and collapse in the wood. The dimensions of the actual, but swollen, water-saturated state should be preserved. Here, the stabilisation of the wooden structure includes the preservation of the shape of the object, which contains information about the manufacturing technique and the function of the archaeological find.

Normally, the evaluation of the dimensional stability of conservation methods is limited to the outer surface of the wood. Until now there are only a few cases that have also considered changes inside wood after conservation using tomographic methods^5,20,23–31^. Among the non-destructive methods currently available, X-ray micro-computed tomography (µCT) is a suitable method that can be used to examine structures inside the entire volume of a sample at various spatial resolutions^32^.

In a prior study, dimensional changes of a range of established and most commonly used conservation methods were investigated on a larger sample series of different wood species using µCT in combination with structured-light 3D scans^33,34^. In this earlier study, µCT was used to examine the samples after conservation for internal defects such as cracks and cell collapse, allowing these to be taken into account when evaluating the conservation result. The results showed that these cracks and collapse occurred much more frequently in the wood samples than previously expected and that in many cases they led to considerable destabilisation. The absence of pertinent information regarding the condition of the samples prior to conservation constituted a significant limitation. Consequently, it was not possible to determine with any certainty whether cracks or collapse were already present prior to conservation.

In this study, the condition of the samples before conservation is also considered when evaluating the conservation result. For this purpose, the three-dimensional structure of the samples was documented and analysed before and after conservation. Due to the difficulty to visualize the structure of wet wood with µCT^33^, the samples were also measured with magnetic resonance imaging (MRI) in the wet condition. MRI is a suitable technique for the visualisation of wet materials and there are different examples for the successful application of this method for the analysis of the structure of WAW^23,35–38^.

In the previous studies^5,33^, alcohol-ether resin, melamine formaldehyde (Kauramin 800), lactitol/trehalose, freeze-drying of polyethylene glycol (PEG)-impregnated woods (one-step with PEG 2000 (PEG1), two-step with PEG 400 and 4000 (PEG2) and three-step with PEG 400, 1500 and 4000 (PEG3), saccharose and silicone oil were investigated. It was decided that a modification to the selection of methods would be necessary, owing to the results obtained and the practicability of the methods: The PEG3 and silicon oil treatments were excluded from the study; however, PEG 2000 with subsequent air-drying (PEG4) was incorporated into the experimental design due to its significant practical relevance in the conservation of large objects where a freeze-drying facility is not available^39–41^.

The aim of this study is to evaluate the dimensional stabilisation of these established and most commonly used conservation methods on a series of samples from different wood species. In addition to the volume changes after conservation, MRI and µCT will enable, for the first time, the quantitative recording of changes and damage in the wood structure, which can be incorporated into the evaluation of the conservation success.

## Materials and methods

### Samples

**Fig. 1:**
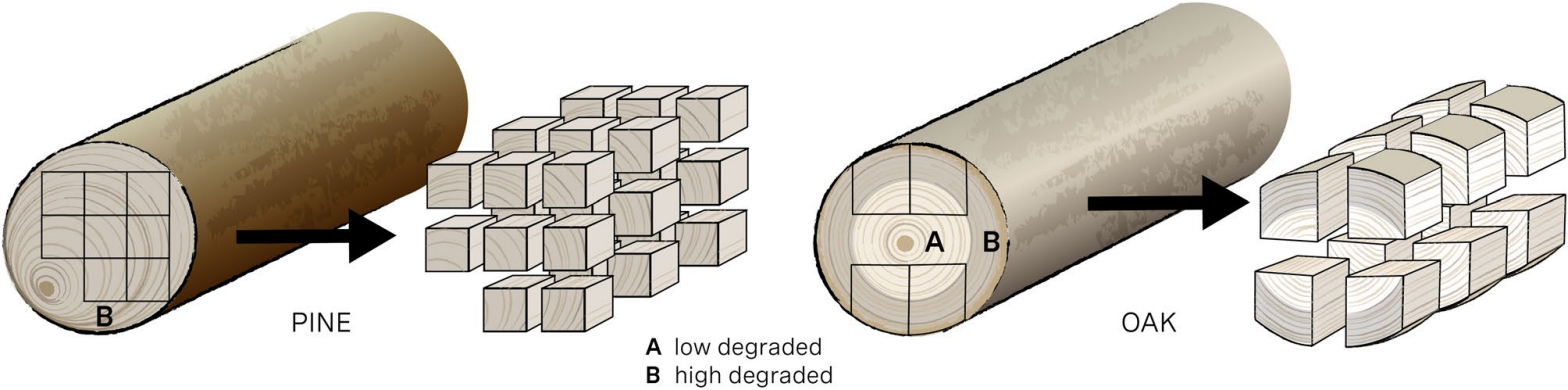
Two piles were cut into cubes with a size of 3 × 3 × 3 cm (pine) and 3 × 3 × 4 cm (oak) respectively (©LEIZA/M.Ober).

Conservation methods must meet a vast number of different requirements so that excellent results are achieved. They should work in different wood species, like softwood and hardwood and on different states of degradation and degradation patterns. In order to test the broad range of challenges of the conservation methods, the following test materials from two different archaeological sites were selected: a heavily degraded pine *(Pinus sp.)* pile (no. 6) from Oberstimm, Bayern, Germany, and an oak *(Quercus sp.)* pile with a medium degree of degradation from Usedom, Mecklenburg-Vorpommern, Germany. This selection reflects both the state of degradation as well as anatomical differences between softwood and hardwood. It was necessary to divide the piles into small cubes (Fig. 1) because the duration of the treatment should not exceed one year. A small sample size reduces both the diffusion time and the drying time. In addition, a high resolution was to be achieved with µCT and MRI in the planned subsequent examinations in order to be able to precisely record the wood structure and the defects it contains. The resolution achieved by the µCT measurements depends directly on the size of the samples^42^. The pile (pine) from Oberstimm was divided into 15 individual discs, which were then subdivided according to the cutting plan (Fig. 1). The pile (oak) from Usedom was divided into 30 individual discs and subdivided in radial direction. When selecting the 40 samples from each pile, care was taken to ensure that the resulting subsamples exhibited a comparable anatomical structure and maximum water content so that both the anisotropic character of the wood and the condition were considered^10^.

The state of preservation of the samples was determined physically. For this purpose, the maximum water content (U_max_, %), the basic density (BD, g/cm³) and the residual basic density (RBD, %) of the wood samples were calculated and classified.

U_max_ is defined as the water present in the sample compared to the absolute wood dry matter^6^. It was determined non-destructively, see formula 1, using the mass of the water-saturated wood and, with the aid of Archimedes’ principle, the mass of the dry wood^43^.

1 $$\:{{U}_{max}}= \frac{{mass_{wet wood}}\:-\:{3\:\cdot\:{mass_{submerged}}}}{3\:\cdot\:{mass_{submerged}}}\:\cdot\: 100\:{\left[{\%}\right]}$$

The state of preservation was classified according to a simple scheme from de Jong^44^, which is based on the maximum water content (U_max_). The pine samples showed wood that was *“very soft*,* no hard core*,* almost no remaining cellulose”* which is classified as 1. The oak samples presented state 2 where *„only a small amount of hard core”* remained.

The water content depends on the void volume in the wood, which is inversely proportional to the basic or conventional density (BD, standard ISO 13061) of the wood in question^3,41,45,46^. The density of the cell wall substance is usually assumed to be 1.5 g/cm³^6,47^. This results in the formula 2^41^.

2 $$\:BD=\frac{100}{\left(66.7+{U}_{max}\right)}\:\:\:\:\left[\frac{g}{{cm}^{3}}\right]\:$$

In order to ascertain the extent of degradation of the wood, the residual basic density (RBD, %) was calculated, representing a ratio between the measured BD of the archaeological material and the average basic density of fresh wood of the same species^6,19,45,48^:

3 $$\:RBD=\frac{BD}{BD\:\left(fresh\:wood\right)}\cdot\:100\:\left[{\%}\right]$$

To ensure the comparability of the samples in the evaluation of the conservation methods, it was necessary to select only those samples which were of a similar condition. Consequently, attention was devoted to ensuring that the resulting subsamples exhibited comparable levels of degradation. The samples had a degree of degradation of (a) pine U_max_ 504 +/-39% (de Jong 1) and a BD of 0.176 +/- 13 g/cm³ assuming a RBD of 42% +/- 3, and (b) oak U_max_ 274 +/- 40% (de Jong 2^44^ and a BD of 0.297 +/- 30 g/cm³ assuming a RBD of 53 +/- 5%.

For this study, the following conservation methods were selected, which are discussed in literature. Alcohol-ether-resin (AlEt)^49,50^, melamine-formaldehyde (Kauramin 800, K800)^51,52^, lactitol/trehalose (LaTr)^53^, saccharose (Sac)^54^ and polyethylene glycol (PEG) with subsequent freeze-drying. PEG treatment followed either a one-stage process with PEG 2000 (PEG1)^55,56^, or, to understand the impact of low molecular weight PEG, a two-stage treatment was selected using PEG 400 and PEG 4000 (PEG2)^19^. In addition, PEG 2000 and air-drying was used to analyse the impact of freeze-drying (PEG4) (Table 1).

**Table 1:** Overview of conservation methods.

Conservation method	Institutions and short descriptions of the methods
Alcohol-ether-resin (AlEt)	Institution: Swiss National Museum, Zürich, Switzerland Treatment: Water progressively replaced by ethanol starting at 85% ethanol increasing concentration ever three weeks until 100% reached. Ethanol progressively replaced by diethyl ether, starting at 70% raising the concentration every there weeks until pure ether present. Three month impregnation in resin-diethyl ether solution. Drying in a vacuum vessel. Impregnation solution: 16.1% dammar, 6.4% rosin, 3.2% castor oil, 3.2% castor oil blown, 0.47% PEG 400 and 70.7% diethyl ether (w/w)
Kauramin 800 (K800)	Institution: Leibniz-Zentrum für Archäologie, Mainz, Germany Treatment: Cleaning in deionized water. Bath impregnation for ca. three weeks at room temperature. Curing of the impregnated wood in a heating cabinet at 60 °C. Afterwards air-drying in PE bags at room temperature over a period of four and a half months. The samples were removed daily (1 h) from the PE bag and weighed until the weight was constant. Impregnation solution: 25% Kauramin 800 solution (72 L resin + 210 L deionised water, 3.6 L urea, 7.2 L triethylene glycol)
Lactitol/trehalose (LaTr)	Institution: Leibniz-Zentrum für Archäologie, Mainz, Germany Treatment: Starting with 30% concentration. Increasing monthly in 10% steps up to 70%. Bath temperature 55 °C. After removal from the bath, the surfaces were dusted with crystalline lactitol monohydrate and dried in a heating oven at 60 °C over a period of one week. After drying, the surface was cleaned by dabbing with a damp cloth. Impregnation solution: Lactitol-trehalose solution (9:1) 30–70%. Addition of biocide if necessary (0.2% (v/v) Parmetol K6)
Polyethylene glycol (PEG 2000) one-step and freeze-drying (PEG1)	Institution: Leibniz-Zentrum für Archäologie, Mainz and Archäologische Staatssammlung, Munich, Germany Treatment: Starting with a 10% PEG 2000 solution. Increasing the concentration in 10% steps up to 40% at room temperature at intervals of eight weeks. Washing of the wood and wrapping in cellulose tissues. Deep-freezing to − 41 °C, freeze-drying in a cooled chamber (approx. − 32 °C) within 21 days. Surface cleaning with a soft brush and ethanol. Impregnation solution: 10–40% PEG2000 in deionised water
Polyethylene glycol (PEG 400 and 4000) two-step and freeze-drying (PEG2)	Institution: Leibniz-Zentrum für Archäologie, Mainz and Archäologische Staatssammlung, Munich, Germany Treatment: Starting with a 10% PEG 400 solution, the concentration was increased after eight weeks with 10% PEG 4000. After a further eight weeks, another 10% PEG 4000 were added. Washing of the wood and wrapping in cellulose tissues. Deep-freezing to − 41 °C, freeze-drying in a cooled chamber (approx. − 32 °C) within 21 days. Surface cleaning with a soft brush and ethanol. Impregnation solution: PEG-solution in deionised water (ca. 10% PEG 400 and ca.20% PEG 4000). The final concentration of the PEG 400 and PEG 4000 solution depends on the degradation of the wood and was calculated individually for each sample from the PEGcon program from the Canadian Conservation Institute (CCI)
Polyethylene glycol (PEG 2000) and air-drying (PEG4)	Institution: Leibniz-Zentrum für Archäologie, Mainz, Germany Treatment: Start with a 10% PEG 2000 solution. Increase the concentration up to 50% at room temperature in 10% steps at intervals of eight weeks. Then, air-drying at 53% relative humidity at room temperature over a period of 5 months. Impregnation solution: 10–50% PEG 2000 in deionised water
Saccharose (Sac)	Institution: Leibniz-Zentrum für Archäologie, Mainz, Germany Treatment: The solution was raised in 10% steps every two weeks from 10% up to 60% saccharose solution at room temperature. Slow, controlled air-drying in micro perforated bags over a period of two and half months. Removal of crystallized sugar residues from the surface with a damp sponge. Impregnation solution: Aqueous sucrose solution 10–60%. If necessary, biocide addition composed of 0.2% (v/v) Parmetol K6 and 0.5% Quartasept plus

### Magnetic resonance imaging

MRI measurements were performed on a small animal scanner with a magnetic field strength of 14.1 T (Magnex Scientific, Oxford, UK), equipped with an RRI (Resonance Research Inc., Billerica, MA, USA) gradient, which is able to reach a gradient strength of 1 T/m within 189 µs. The total bore size of 26 cm was reduced to a usable diameter of 12 cm inside the gradient. The system was interfaced to a Bruker Avance 3 console (Bruker BioSpin, Ettlingen, Germany) running ParaVision 6.0.1 (https://www.bruker.com). The samples were inserted into an air-tight plastic container to avoid dehydration and placed into a home-built birdcage transmit/receive coil with an inner diameter of 6 cm. After basic adjustments and shimming, a high-resolution multi-spin-echo imaging sequence was used to acquire 70 slices with a thickness of 0.5 mm and a spatial resolution of 50 × 50 µm^2^ over a field-of-view of 38.4 × 51.2 mm^2^. Four echoes per phase encoding step with echo times of 10 ms, 20 ms, 30 ms and 40 ms were recorded, of which only the first two were reconstructed and added to obtain the final images. With a repetition time of 3.3 s, the entire measurement took 56 min.

### X-ray micro-computed tomography

In addition to the MRI measurements of the wet samples, also µCT measurements were performed before conservation in an industrial XCT scanner (d2 from Diondo, Hattingen, Germany). The devices feature an X-ray source (XWT-225 TCHE + from X-ray works, Garbsen, Germany) in high power mode and an accelerating voltage of 120 kV and a tube current between 167 and 250 µA with a 1 mm aluminium prefilter. The wood samples were mounted in a sample holder and placed in the sample chamber. The sample was rotated 360° in continuous mode during the acquisition. The radiographic projections were recorded with a 4343 DX-I X-ray detector (Varex, Salt Lake City, U.S.A.), with a pixel size of 139 μm. The distance between the X-ray source and the sample was between 133 and 158 mm and the distance between the X-ray source and the detector was between 700 and 850 mm, giving a magnification between 5.3 and 5.4 and a nominal voxel size between 27 and 29 μm. A total of 2250 projection images were acquired during the sample rotation of 360° at equal angular step. The conserved and dried samples were also measured by setting the X-ray source to high power mode. The operation voltage was 60 kV and a filament current of 300 µA with a 1 mm aluminium pre-filter was chosen. Here, the distance between the X-ray source and the sample was between 70 and 140 mm and the distance between the X-ray source and the detector was between 450 and 600 mm, resulting in a magnification between 4.3 and 6.4 and a nominal voxel size between 23 and 35 μm. A total of 2800 projection images were acquired during the sample rotation of 360°. The resulting projections were converted into a 3D image stack of approx. 3000 × 3000 × 3000 voxels using the CERA 7.0 reconstruction software (https://www.oem-products.siemens-healthineers.com) based on the filtered back projection Feldkamp algorithm from Siemens^57^.

### Evaluation of magnetic resonance imaging and X-ray micro-computed tomography

**Fig. 2:**
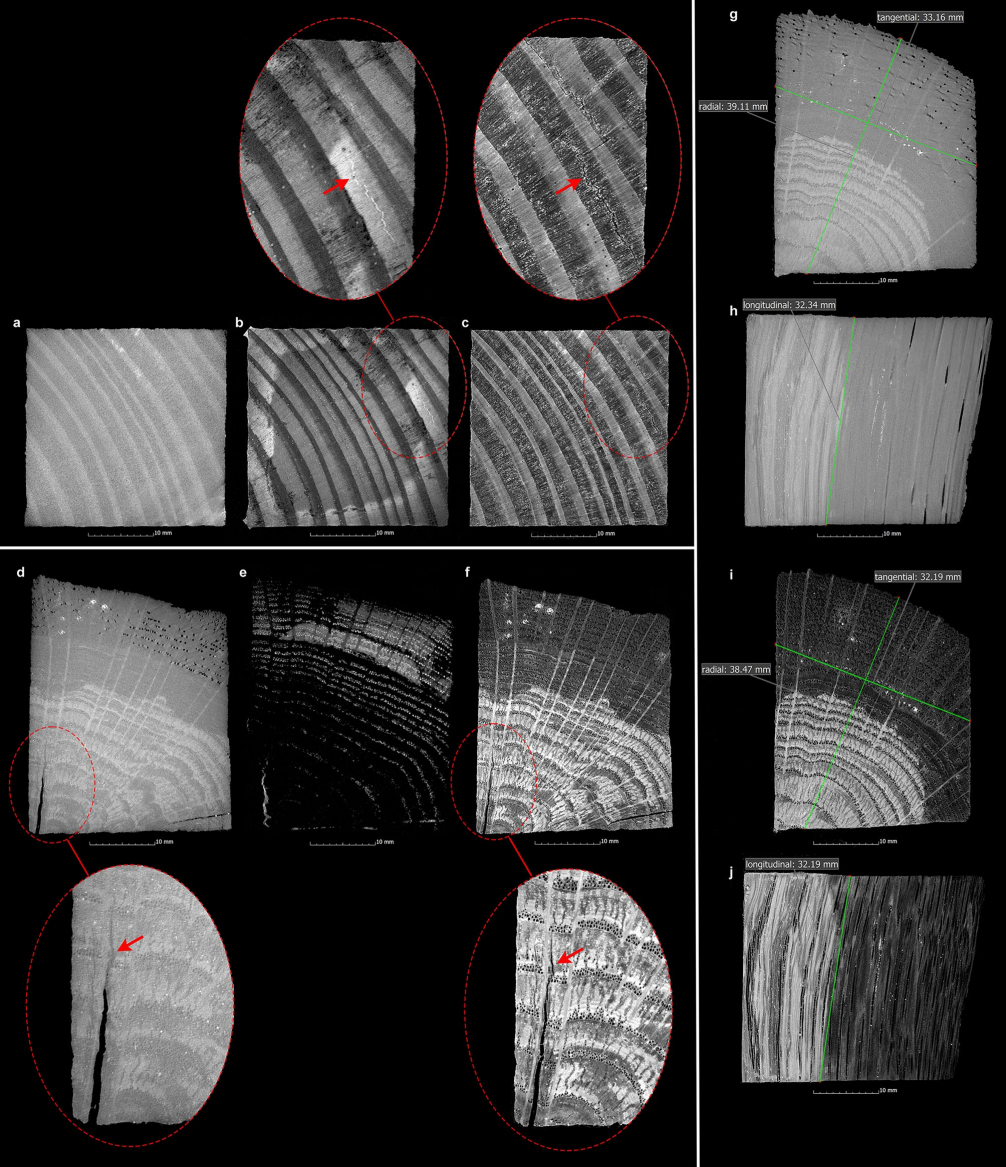
Cross-sectional images of a pine wood sample (Pi-AlEt-4) in the pre-conservation state (top left) with µCT (**a**) and MRI (**b**) as well as after conservation with the alcohol-ether method with µCT (**c**). Highlighted details show a crack after conservation with µCT (**c**), which could only be detected with MRI (**b**) and not with µCT in the pre-condition. The cross-sectional images of an oak sample (Oa-AlEt-1, bottom left) similarly show the condition before conservation with µCT (**d**) and MRI (**e**) as well as after conservation with the alcohol-ether method with µCT (**f**). Highlighted details show a crack after conservation with µCT (**f**), which can already be determined in the pre-condition with both µCT (**d**) and MRI. On the right the measurements of the wood anatomical directions (tangential, radial and longitudinal) are shown before (**g**, **h**) and after (**i**, **j**) conservation of an oak sample (Oa-AlEt-3).

In order to record the condition of the samples and the changes in the wood structure after conservation, the MRI and µCT data of the untreated wet samples were compared with the µCT data of the conserved samples (Fig. 2a-f). This comparison showed that the state of preservation of the unconserved samples has a considerable influence on the quality of the data of the different measurement methods. For instance, the wood structure in the samples of pine, which are poorly preserved, is very easily identifiable in the MRI data due to the high water content (Fig. 2b). In contrast, in the oak samples, which have better preserved areas with lower water content (the inner area of the pile, Fig. 1), the structure is difficult, if not impossible, to recognize (Fig. 2e). The varying states of preservation exhibited by the oak samples are also discernible in this context. The different degrees of degradation are evident in the MRI data (Fig. 2e), which can be further substantiated by the findings from the µCT measurements (Fig. 2d, f). The µCT provides a greater quantity of information on the better preserved oak samples than the MRI. Conversely, the wood structures of the poorly preserved pine samples are less discernible due to the high water content present in the µCT data (Fig. 2a) in comparison to the MRI (Fig. 2b) data. This influence of the water content in WAW on MRI and µCT was also reported^37^.

To record changes in volume and surface of the samples, the data was processed with VGSTUDIO MAX 3.4 (https://volumegraphics.hexagon.com). Due to the challenges posed by incomplete information and the presence of surrounding water, which hindered a precise localisation of the sample’s surface within the MRI data, µCT data from before and after conservation were utilised for surface determination. However, the MRI data were used in conjunction with the µCT data to ascertain the presence of cracks, collapse or other defects in the wood prior to conservation. Notably in the case of the wet pine samples, the MRI data yielded information that was not obtainable through µCT (Fig. 2a-c).

The surface determination in the µCT of the very heterogeneous material was done manually for each sample with the different segmentation tools of the software. Afterwards the volume and surface values were calculated.

The evaluation of the conservation methods was conducted by determining the dimensional stability, which was measured using volume data derived from the surface of the entire sample. Additionally, the values of the individual wood anatomical directions (tangential, radial and longitudinal) before and after conservation were measured in the µCT data using the measuring tool in VGSTUDIO MAX 3.4. (Fig. 2g-j). To evaluate the volume changes of the samples, the data from µCT was used to calculate the shrinkage (S) from the volume before (V_wet_) and after (V_dry_) conservation^58^:

4 $$\:S=\frac{{V}_{wet}-{V}_{dry}}{{V}_{wet}}\:\times\:100\:\left[{\%}\right]$$

The shrinkage for the anatomical directions tangential (S_tan_), radial (S_rad_) and longitudinal (S_long_) was calculated equally. The anti-shrink efficiency (ASE) was determined from the shrinkage of a non-conserved control sample (S_o_) and the shrinkage of the conserved sample (S_con_)^59^:

5 $$\:ASE=\frac{{S}_{0}-{S}_{con}}{{S}_{0}}\:\times\:100\:\left[{\%}\right]$$

An ASE of 100% indicates excellent conservation, whereas an ASE of 0% is equivalent to the outcome of air-drying. An ASE of 75% is generally considered to be acceptable^22,60^. By using ASE, a statistical evaluation and comparison of conservation results is possible. In addition, the ASE in the anatomical directions tangential (ASE_tan_), radial (ASE_rad_) and longitudinal (ASE_long_) shows which direction of the wood anatomy is stabilised to what extent^61–64^. In order to obtain a more detailed perspective on the deformation that occurs during the conservation process, the surface determination of the µCT data was used for a nominal-actual comparison of the samples before and after conservation. This was processed for each sample using the VGSTUDO MAX 3.4 geometry analysis module. This data was exported into a surface mesh STL data and is available for download on Zenodo (10.5281/zenodo.16535029).

**Fig. 3:**
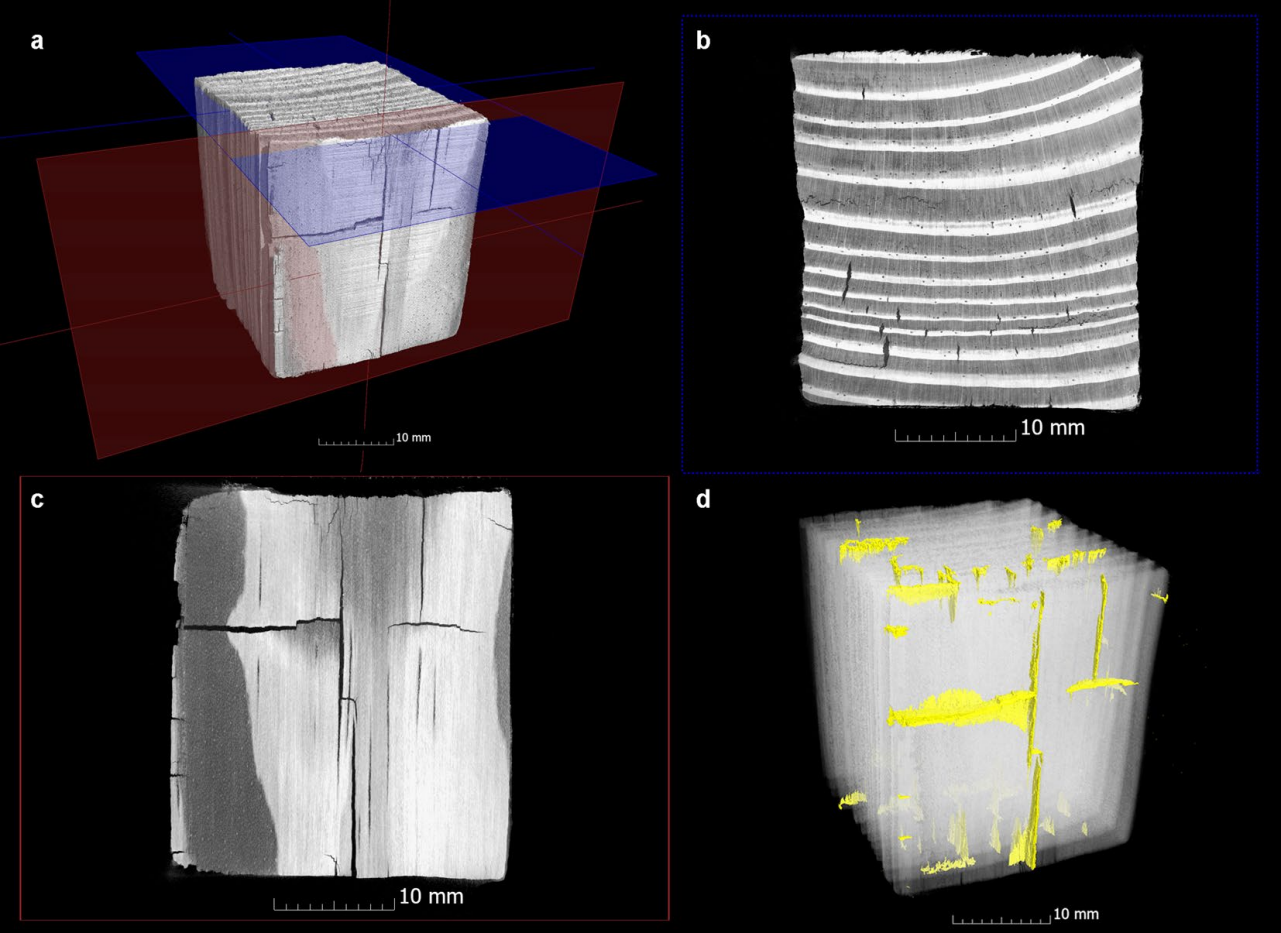
CT visualisation of the cracks in a pine sample (Pi-K800-2) conserved with Kauramin 800. The position of the cross section (**b**) and longitudinal section (**c**) are shown as blue and red planes in the 3D visualization (**a**). The 3D visualization (**d**) shows the cracks in yellow colour, which in this case only occur in the outer areas of the sample. In the cross-section (**b**), radial and tangential cracks can be seen, whereby collapse is also visible here in isolated cases. In the case of Kauramin 800, there is a transitional area between cracks and collapse and a clear separation is not always possible. In the longitudinal section (**c**), transverse cracks can be seen alongside radial cracks.

**Fig. 4:**
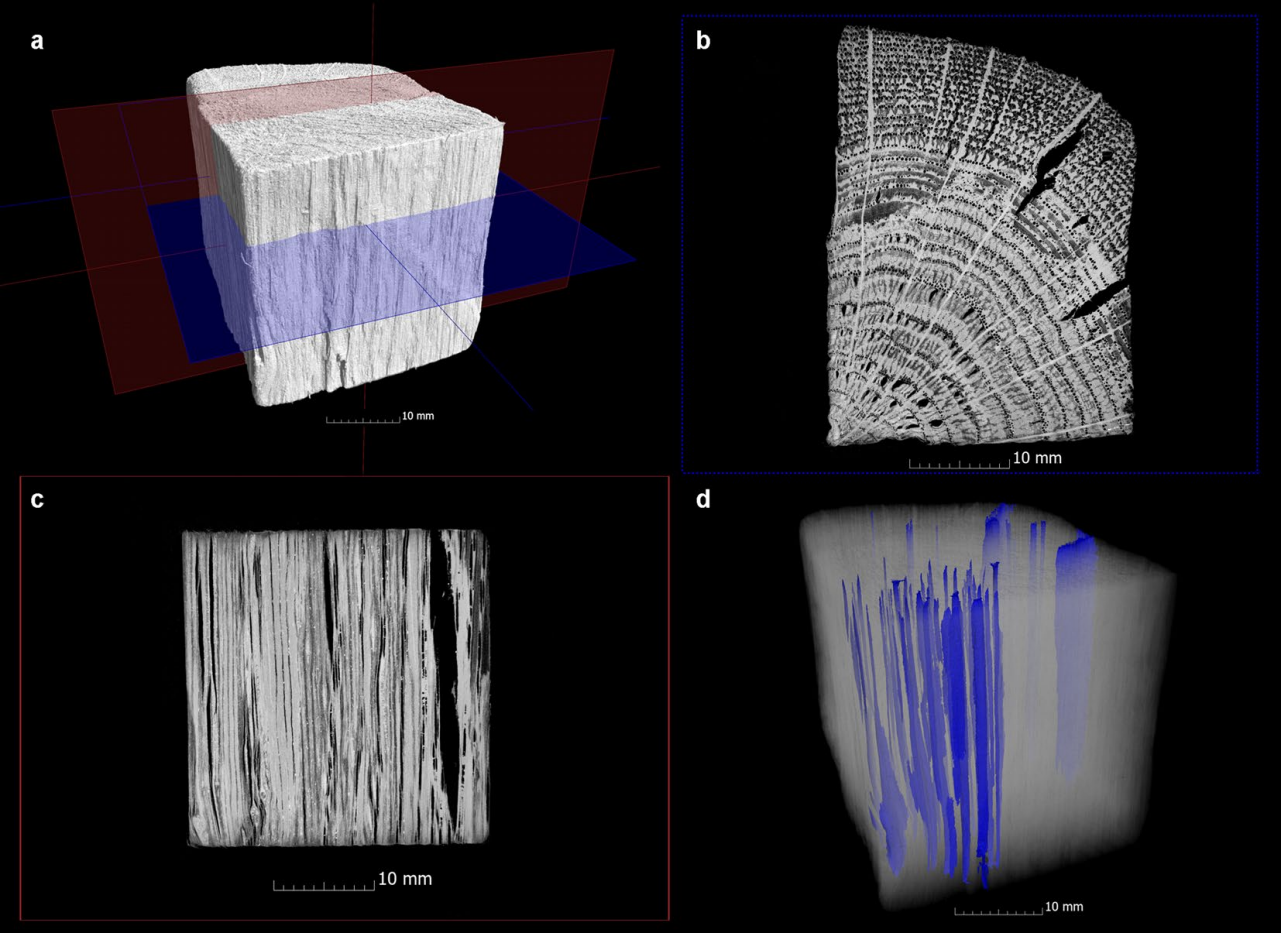
CT visualisation of the collapse in an oak sample (Oa-Sac-3) conserved with Saccharose. The position of the cross section (**b**) and longitudinal section (**c**) are shown as blue and red planes in the 3D visualization (**a**). The 3D visualization (**d**) shows the collapse in blue colour. While in better preserved wood the collapse is smaller and limited to annual rings but occurs more frequently, in poorly preserved wood there are fewer but larger areas that have collapsed.

A visual inspection of the data was conducted to ascertain the presence of defects. These were then compared with the data from the previous condition (Fig. 2a-f). The defects under investigation included cracks (Fig. 3) that ran radially, tangentially or transversely as well as collapses that either only occurred in individual growth rings or spread over a larger area (Fig. 4). In contrast to narrow, long cracks, collapses are spindle-shaped cavities in a radial direction, which were created by the breakdown of the cell walls^10,34^. The newly formed cracks and collapses inside the samples were segmented separately with VGSTUDIO MAX 3.4 in order to analyse the extent to which cracks and collapses occurred during conservation. To obtain a quantitative understanding of the size of these cavities inside the samples, the volumes and surfaces of existing cracks and the collapse were determined from the µCT data. From these values the sphericity ѱ of the cracks was calculated to get a better quantitative overview of their shape:

6 $$\uppsi = \frac{{\sqrt[3]{{36\pi V^{2} }}}}{{A_{O} }}$$

The sphericity ѱ relates the shape of a body based on its volume (V) and its surface (A_O_) to the smallest possible surface of a sphere of the same volume^65^. The value of the sphericity ѱ for a sphere is 1. The lower a value of sphericity ѱ is for a shape, the larger the surface area is compared to a sphere. In addition, the maximum width and length of the cracks in the µCT data were measured.

## Results and discussion

### Volume changes

The volumes of the samples before and after conservation were determined from µCT data. Subsequently, alterations in the volume of all samples were calculated. In addition to the changes of the outer surface (S_surface_), the loss of volume due to cracks and collapse inside the samples (S_cavities_) was considered for the different wood species and conservation methods. The calculation of shrinkage (S, %) was conducted on the basis of the total loss of volume. (Table 2).

The pronounced shrinkage of the unconserved, air-dried reference samples in comparison to the conserved samples is evident, with the unconserved oak samples exhibiting a shrinkage exceeding twice that of the pine samples. This difference is present despite the poorer state of preservation of the pine samples. This observation is consistent with the behaviour of contemporary wood species, wherein higher density is accompanied by greater volume shrinkage or swelling capacity^10^. Within the conserved samples of both test series, the alcohol-ether resin (AlEt) method showed the smallest change and the samples conserved with PEG with subsequent air-drying (PEG4) revealed the largest change in volume on average.

When considering the different drying processes of the methods, a comprehensive analysis of the experimental data of both types of wood reveals that the air-dried wood samples (Kauramin 800, lactitol/trehalose, PEG4 and saccharose) show a greater volume loss than the freeze-dried (PEG1 and PEG2) or solvent-dried (alcohol-ether resin) samples. Only the volume loss of 5.9 ± 1.5% for lactitol-trehalose (air-dried) in the pine samples is slightly lower than the 6.7 ± 0.8% for PEG1 (freeze-dried). It is evident from the findings of this study that cavities formed inside the samples play a lesser role in pine samples than in oak samples. Furthermore, it is noteworthy that the volume loss due to cavities in the oak samples conserved with Kauramin 800 is greatest at 1.9%, while the alcohol-ether method is the only one that shows no cavities after drying.

**Table 2:** Shrinkage (S) mean values (*n* = 5) ± standard deviations for the investigated conservation methods.

	Conservation methods							
	Air	AlEt	K800	LaTr	PEG1	PEG2	PEG4	Sac
**Pine (*n*** **= 5)**								
S (%)	26.2 ± 1.9	4.1 ± 0.8	9.2 ± 2.0	5.9 ± 1.5	6.7 ± 0.8	4.5 ± 0.3	12.4 ± 5.7	7.6 ± 1.0
S_ surface_ (%)	26.1 ± 1.9	4.1 ± 0.8	9.0 ± 1.9	5.9 ± 1.5	5.9 ± 0.5	4.4 ± 0.3	12.4 ± 5.7	7.3 ± 1.1
S_ cavities_ (%)	0	0	0.2 ± 0.1	0	0.8 ± 0.5	0.1 ± 0.0	0	0.3 ± 0.2
S_ tan_ (%)	24.4 ± 4.3	1.8 ± 0.5	4.4 ± 1.5	2.2 ± 0.8	1.7 ± 0.7	1.3 ± 0.8	7.7 ± 4.6	4.0 ± 0.3
S_ rad_ (%)	6.4 ± 5.6	1.3 ± 0.9	1.8 ± 0.6	1.7 ± 0.7	2.5 ± 0.2	1.4 ± 0.2	0.8 ± 1.4	1.9 ± 0.6
S_ long_ (%)	4.4 ± 1.7	1.5 ± 0.9	2.7 ± 0.7	1.3 ± 0.4	2.0 ± 0.3	1.4 ± 0.3	0.9 ± 0.5	1.4 ± 0.6
**Oak (*n*** **= 5)**								
S (%)	55.2 ± 4.7	6.1 ± 0.5	11.1 ± 1.9	16.5 ± 0.6	7.5 ± 0.7	6.7 ± 2.0	25.2 ± 3.0	10.2 ± 5.2
S_ surface_ (%)	53.3 ± 5.2	6.1 ± 0.5	9.2 ± 3.0	14.8 ± 1.2	6.4 ± 0.7	6.1 ± 2.0	24.2 ± 2.8	8.4 ± 5.8
S_ cavities_ (%)	1.3 ± 0.6	0	1.9 ± 1.2	1.7 ± 0.9	1.1 ± 0.4	0.6 ± 0.2	1.0 ± 0.3	1.7 ± 1.1
S_ tan_ (%)	23.2 ± 4.3	3.4 ± 0.6	3.7 ± 1.4	7.0 ± 2.0	2.0 ± 1.3	2.9 ± 0.9	12.6 ± 1.5	5.6 ± 1.3
S_rad_ (%)	14.7 ± 1.5	1.5 ± 0.2	3.0 ± 2.7	1.8 ± 1.9	2.3 ± 1.3	2.1 ± 0.6	4.5 ± 1.2	1.6 ± 1.5
S_ long_ (%)	8.0 ± 3.7	0.9 ± 0.3	2.0 ± 0.7	1.4 ± 0.5	0.8 ± 0.5	0.9 ± 0.3	1.4 ± 0.3	0.4 ± 1.4

In the unconserved reference samples, the greatest shrinkage was observed in the tangential direction (Table 2). This was followed by radial shrinkage and the least contraction occurred in the longitudinal direction, which corresponds to the behaviour of contemporary wood and is influenced by a number of mechanisms, including the orientation of micelles, fibrils and wood fibres and rays^10^. The conserved oak samples also follow this tendency, with the exception of PEG1. In the pine samples, it was found that only those which had been conserved with lactitol/trehalose and saccharose exhibited this particular behaviour. The other specimens do not follow this tendency of shrinkage (tangential > radial > longitudinal), with the exception of PEG1 and PEG2, most of the shrinkage also occurred in the tangential direction but followed by the longitudinal direction. The PEG1 and PEG2 conserved samples, on the other hand, shrunk more in the radial and longitudinal than in the tangential direction.

### Dimensional stabilisation provided by the conservation methods

**Fig. 5:**
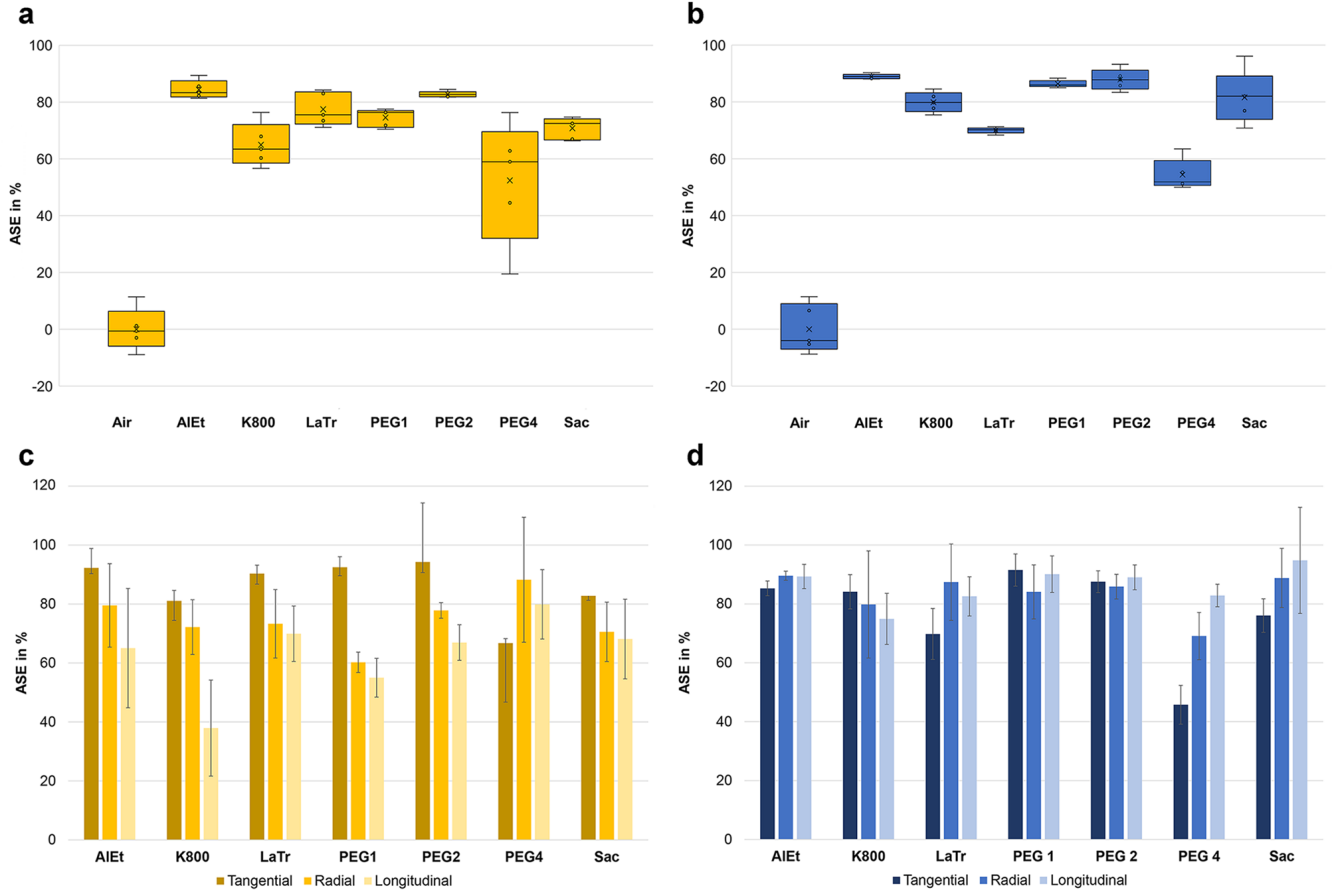
ASE in dependence of the conservation method: (**a**) ASE (volume) of pine samples (**b**) ASE (volume) of oak samples (**c**) ASE of pine samples in tangential, radial and longitudinal direction, (**d**) ASE of oak samples in tangential, radial and longitudinal direction.

The ASE was calculated from the total shrinkage (S), which included the cavities present in the samples (Fig. 5). Among all the conservation methods tested, alcohol-ether resin was found to have the best ASE values. This applies both to the pine samples with an average ASE value of 84.4 ± 3.2% and to the oak samples with an average ASE value of 89.9 ± 0.9% (Fig. 5a). The efficiency of the method is further validated by the outcomes of other test series^33,34,66^, especially for deciduous woods^67^. This may probably be explained foremost by the very gentle solvent-drying^21,50^. Nevertheless, it is notable that there exist test series for which this method exhibits inferior performance. This is probably particularly true for heavily degraded softwoods and can likely be explained by the shrinking of the S2 layer within the secondary cell wall^5,18,66,68,69^.

The majority of studies on the subject have shown that the use of PEG methods followed by freeze-drying leads to satisfactory stabilizing results^18,59,70,71^, which is in accordance with the results of the present study. The two PEG treatments with subsequent freeze-drying both demonstrated effective performance, with PEG2 exhibiting superior ASE values (82.7 ± 1.1% for pine and 87.9 ± 3.7% for oak) in comparison to PEG1 (74.5 ± 3.2% for pine and 86.4 ± 1.3% for oak).

**Fig. 6:**
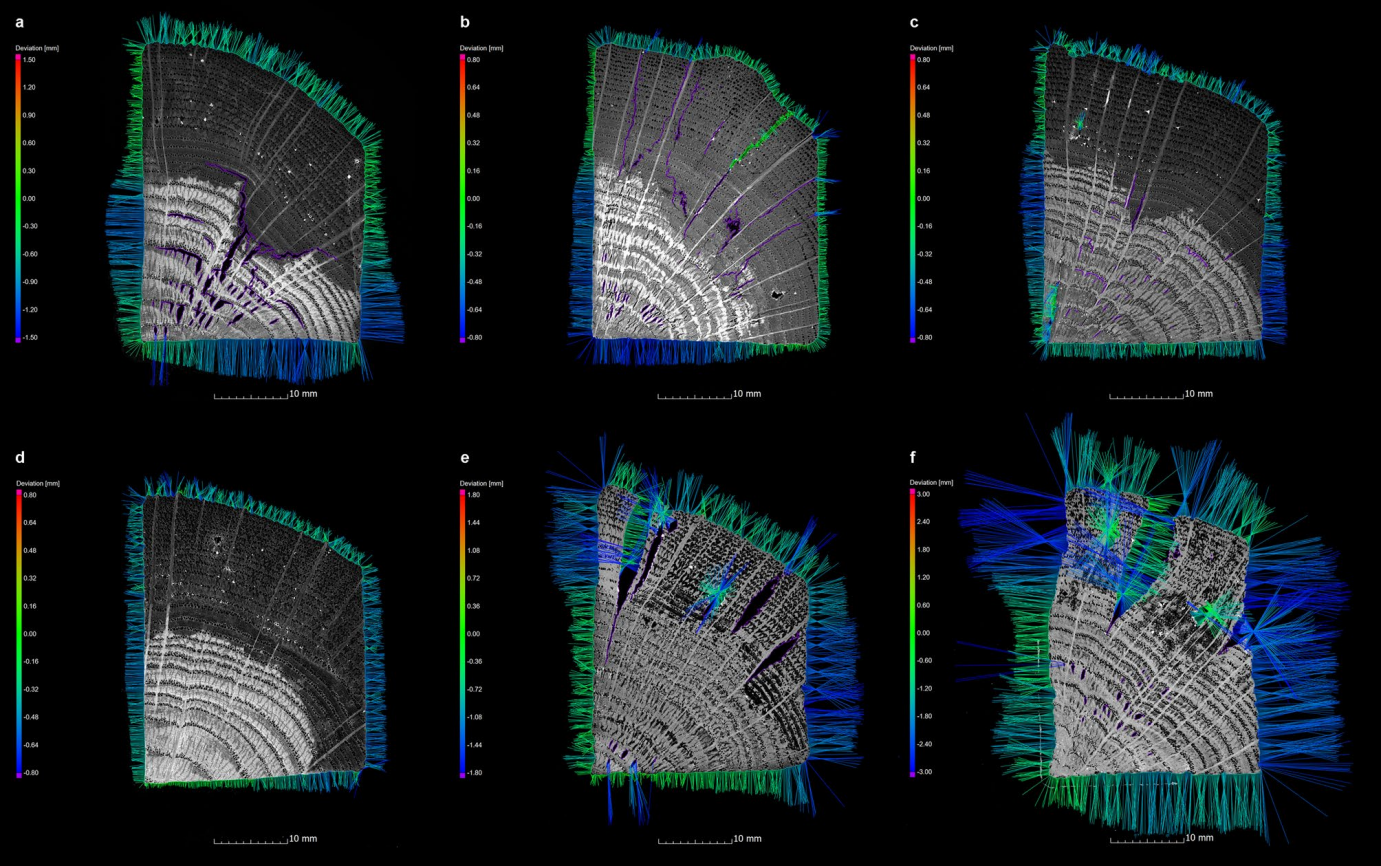
Nominal actual comparison of the volume of oak samples before and after conservation with (**a**) Kauramin 800 (sample Oa-K800-5), (**b**) PEG1 (sample Oa-PEG1-2), (**c**) PEG2 (sample Oa-PEG2-3), (**d**) alcohol-ether-resin (sample Oa-AlEt-3), (**e**) lactitol/trehalose (sample Oa-LaTr-2) and (f) PEG4 (sample Oa-PEG4-4).

Lactitol/trehalose has a better ASE of 77.5 ± 5.8% for pine than PEG1, but with a value of 70.0 ± 1.1% for oak, the overall volume stabilisation is worse than that of PEG1. Furthermore, lactitol/trehalose exhibited a substantial standard deviation of 5.8% for the pine samples. This corresponds to earlier studies in which lactitol/trehalose achieved good dimensional stability, particularly in the case of softwoods^20,72^. The study by Hoffmann^41^ yielded less consistent results, a phenomenon that may be attributed to the non-uniform distribution of the sugar alcohols (Fig. 6e). An observation that is also evident on a microscopic scale^5^.

The samples conserved with saccharose showed a less consistent result. However, when both wood species are considered collectively, saccharose also has a better average performance in comparison to lactitol/trehalose. It is noteworthy that the ASE value of 70.8 ± 3.9% for the pine samples is suboptimal, while the superior value of 81.6 ± 9.3% for the oak samples demonstrates a high degree of variability. These inconsistent results confirm earlier studies^73^ and also support the assumption that better preserved woods are more likely to be stabilised with saccharose^74^.

Kauramin 800 achieved very different results for the two wood species with an ASE of 64.9 ± 7.6% for pine and 79.9 ± 3.5% for oak, whereby the large standard deviation in the pine samples is also noticeable. These outcomes do not correlate with the good results previously achieved with this method^18,33,75^.

It is evident that the use of PEG in conjunction with subsequent air-drying (PEG4) has yielded the most unfavourable outcome for both types of wood (52.4 ± 21.6% for pine and 54.4 ± 5.4% for oak). The method demonstrated the highest standard deviation for the pine samples. This high value is attributable to two samples within the five samples conserved with PEG4 whose ASE values of 19% and 76% are significantly divergent. As the samples did not show any specific anomalies in their previous state that could account for such a substantial deviation, it appears that there is a direct correlation with the state of preservation: The sample demonstrating an ASE value of 19% exhibited the highest U_max_ of 541%, thereby reflecting the most significant degree of degradation amongst the five samples conserved with PEG4. Conversely, the sample with an ASE value of 76% had an U_max_ of 471% and is therefore less degraded in comparison. The ASE values for the different anatomical directions of the wood show that PEG4 does not achieve sufficient stabilisation in the case of the pine samples (Fig. 5c), particularly in the tangential direction. As this altered behaviour is the direction in which the main shrinkage occurs, this leads to poor overall stabilisation. PEG4 attains better results in the radial and longitudinal directions compared to all the other methods. However, these other methods demonstrate a consistent pattern in the pine samples, whereby they exhibit the greatest stability in the tangential direction, followed by the radial direction and finally the longitudinal direction. This difference can be attributed to the observation that the shrinkage in the tangential direction is the most significant in the reference samples. Consequently, it is evident that the stabilising effect of the conservation agents is most pronounced in this direction and decreases for the radial and longitudinal directions. In the longitudinal direction, the shrinkage in the reference samples examined here is minimal, which explains why the stabilizing effect is less pronounced in this direction.

The same tendency of shrinkage in the anatomical directions is also present in the oak samples, although it is much less pronounced. With 8% shrinkage in the longitudinal direction, the value for the air-dried reference sample is relatively high. This large deviation also seems to be the reason why the ASE values do not follow such a clear trend as in the pine samples. Another aspect of the volume stabilisation of the oak samples is that there are two different preservation states within the samples. The nominal-actual comparisons clearly demonstrate that there is a deviating behaviour in the volume stabilisation of the different preservation states with the various conservation methods (Fig. 6). Kauramin 800 and the methods with PEG with subsequent freeze-drying (PEG1 and PEG2) clearly show that the better preserved area shrinks more. With the alcohol-ether method, this is less noticeable and both areas are stabilised rather equally well. Both series of oak samples, conserved with lactitol/trehalose and PEG with subsequent air-drying (PEG4), in contrast, show that the more degraded areas shrink more, as is also the case with the unconserved samples. The saccharose conserved oak samples show no discernible uniform tendency.

### Cracks and collapse

**Fig. 7:**
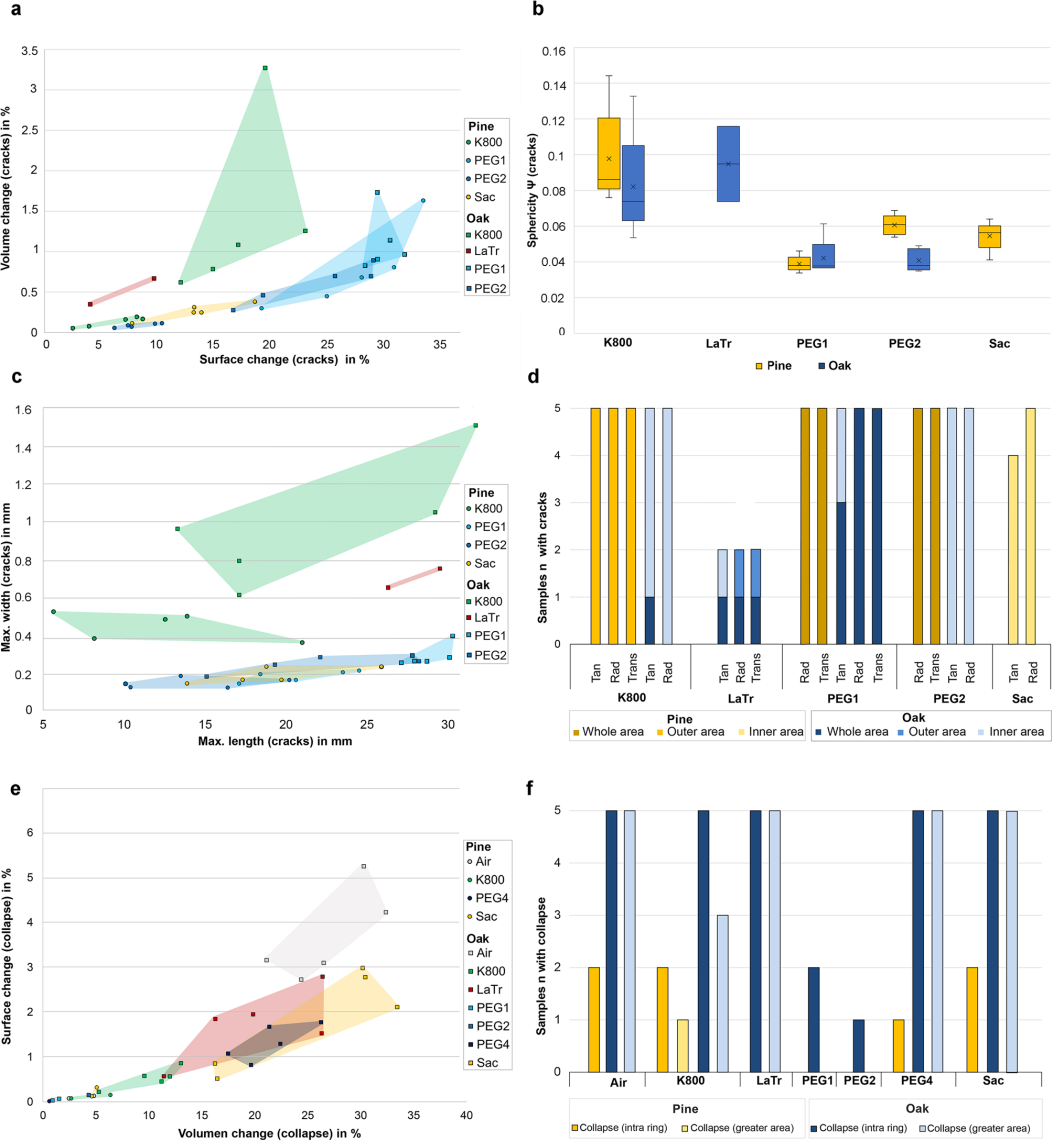
Quantitative evaluation of the cracks and collapse in the samples: (**a**) volume and surface area of the cracks in relation to the sample, (**b**) sphericity of the cracks, (**c**) direction and position of the cracks in the samples, (**d**) maximum width and length of the cracks, (**e**) volume and surface area of the collapse in relation to the sample, (**f**) type and position of the collapse in the samples.

The existing damage patterns inside the samples were divided into cracks (Fig. 3) and collapses (Fig. 4) and the volume and surface area of these two damage patterns were recorded separately. It was initially found that none of this damage could be detected when examining the samples conserved using the alcohol-ether method. In contrast, all other methods showed the formation of cavities due to cracks or collapse after conservation. The Fig. 7a and e give an overview to what extent the cracks and collapse occurred in the samples by giving the volume and surface change in percent in relation to the volume and surface of the whole sample after conservation. The calculated sphericity as well as the measured width and length give an impression of the shape of the cracks (Fig. 7b, c). Figure 7d shows the direction of these cracks and whether they are limited to the outer or inner area of a sample. For the existing collapse, Fig. 7f shows whether this is limited to annual rings (intra-ring) or if a larger area is involved. 

It can be seen from the values of the sphericity (Fig. 7b) and the size (Fig. 7c) that the PEG processes with subsequent freeze-drying (PEG1 and PEG2) tend to have fine and long cracks, which can be attributed to the freezing process as a preparatory step for freeze-drying. The occurrence of collapse is negligible in the context of these methods. Only isolated collapse can be observed in the oak samples.

However, it is evident that there is a higher prevalence of cracks in the PEG1 samples when compared to the PEG2 samples. This difference can be attributed to the addition of the low-molecular-weight PEG 400, which acts as a cryoprotectant^43,76,77^. This finding corroborates the results of the previous study^33^, in which it was hypothesised that the volume expansion of the aqueous PEG solutions, which is known to cause impairment to the wood structure, is mitigated by the low-molecular weight PEG 400^78^.

**Fig. 8:**
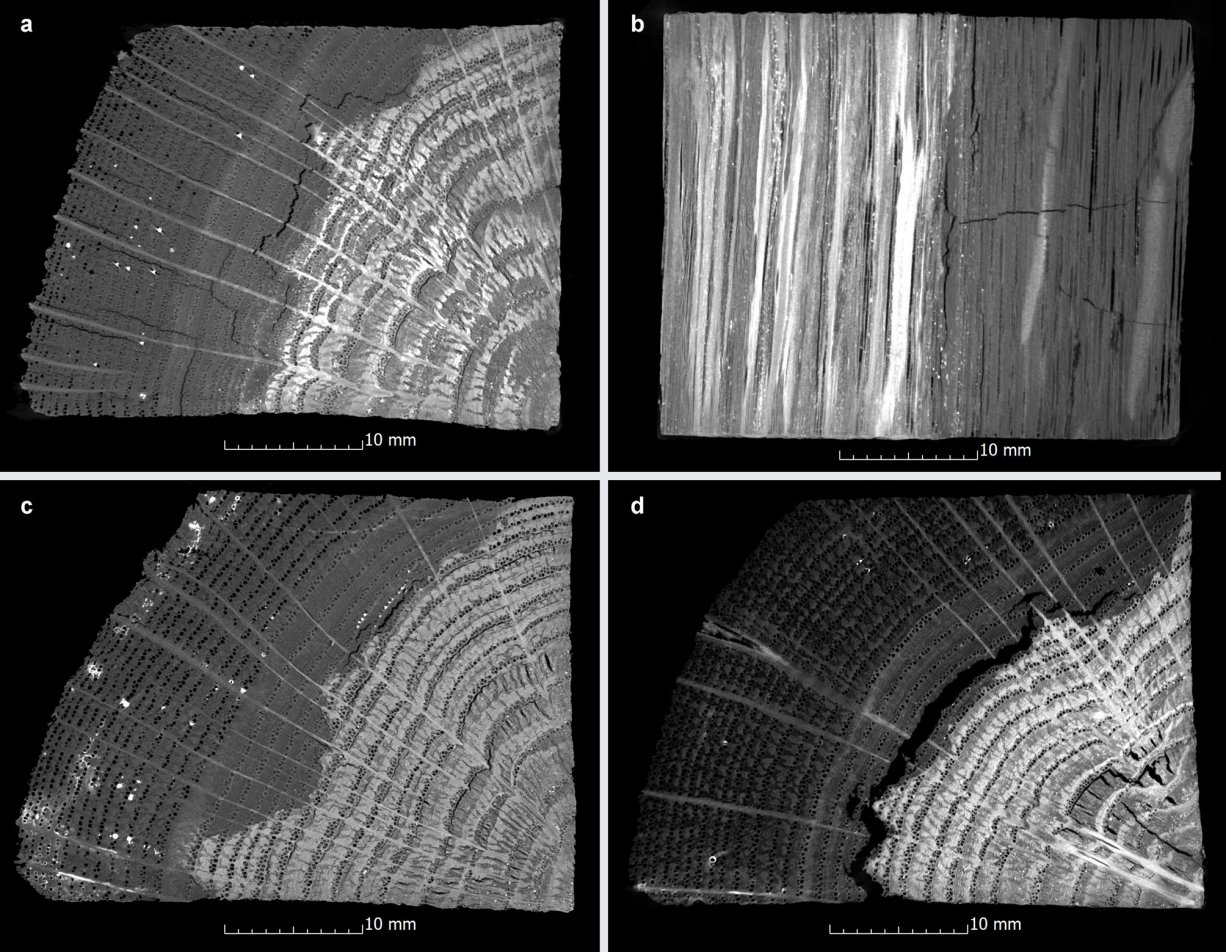
CT images of (**a**) radial, tangential and (**b**) transverse cracks in a PEG1 conserved oak sample (Oa-PEG1-4) as well as radial and tangential cracks in oak samples conserved with (**c**) PEG2 (Oa-PEG2-2) and (d) Kauramin 800 (Oa-K800-4).

For the pine samples conserved with PEG1 and PEG2, it is obvious that all samples show radial and transverse cracks in the entire area, while the oak samples show a different behaviour (Fig. 7d). The PEG1 oak samples show a distribution of cracks in all directions over the whole volume (Fig. 8a, b, transversal only in the poorly preserved wood), while the PEG2 samples only show cracks in the radial and tangential direction in the inner area (8c). The extent to which the number, position and different orientation of cracks found here depend on the type of wood, the condition of the wood and the PEG used requires further examination on a microscopical level.

Another conservation method that shows characteristic cracks in this study, which in this case occur during air-drying, is Kauramin 800: The outer areas of the pine samples show rather small cracks that run in all three anatomical directions. Collapse can only be observed to a small extent in these samples (Fig. 7e). It occurs mainly in the outer area (see Fig. 3) and in wood conserved with Kauramin 800 collapse cannot always be clearly distinguished from cracks. In addition to collapsing cell walls, this damage might also be caused by drying stress^79,80^. Compared to the pine samples, the oak samples showed greater collapse and considerable crack formation. These cracks run radially and tangentially and are also quite large according to the sphericity value. This is confirmed when looking at the cross-sections (Fig. 8d), in which wide tangential cracks run along the annual rings. These cracks are also mostly located in the transition area between poorly and well preserved wood. In this area, considerable stresses appear to occur with this conservation method in the case of two different states of preservation. This damage phenomenon in oak was also shown in the previous study^33,34^. One potential explanation for this damage pattern is the chemical bonding that Kauramin 800 forms especially with the lignin present^81,82^. In this case the composition of the residual cell wall could be decisive because it determines the chemical compatibility of the conservation agent with the decayed wood substance. In totally disintegrated wood, the structure consists of a network of intact middle lamella, an amorphous residual material with lignin and lignin-like substances^83^. Especially the small in-between areas showing intermediate decayed xylem in oak could be a crucial factor which causes the wood to split in this area^7^. Here the strong chemical bonding of Kauramin 800 and lignin as well as the fact that it is stiff and is not easy to deform^66^ may lead to stresses between the different preserved areas. This chemical behaviour would also explain why heavily degraded wood is better stabilised by Kauramin 800 than well-preserved wood^73,81^ which also became clear in this study in the nominal-actual comparisons (Fig. 4a). Here, further analysis would be essential.

Looking at the other conservation agents (lactitol/trehalose, PEG4 and saccharose), which, like Kauramin 800, are based on air-drying, it is initially noticeable that all the pine samples conserved with saccharose show slight cracks in the outer areas in a radial and tangential direction (Fig. 7a-d). The samples conserved with lactitol/trehalose show only isolated cracks, whereby two oak samples stand out with cracks in all three anatomical directions (Fig. 7d). The formation of cracks can occur with lactitol/trehalose due to the crystallisation to lactitol MC trihydrate, as this is associated with an increase in volume^5,84^. Collapse does not occur significantly in any of the methods with air-drying for the pine samples.

A completely different picture was obtained in the oak samples, where considerable collapse was observed in all these conservation methods with subsequent air drying. Most collapse can be detected in the unconserved samples (Fig. 7e). This ranking is followed by Saccharose, lactitol/trehalose and PEG4. Among the methods with air-drying, the oak samples conserved with Kauramin 800 show the least collapse, which is also confirmed by the previous study^33,34^. Here, lactitol/trehalose and saccharose also showed the greatest collapse of the wood structure.

The structural changes observed here went beyond small collapse limited to one annual ring (intra ring), and larger areas also collapsed. Figure 7f gives an overview of the samples in which the collapse occurred and in what form.

It was found that intra-ring collapse took place predominantly in the well-preserved areas of the oak samples, while in the poorly preserved areas it clearly extended over larger areas (Fig. 4). This can be explained by the capillary forces occurring during air drying, which especially the completely disintegrated cells cannot withstand^10,11,80^.

## Conclusion

In this study, the µCT and MRI data from test series with two different wood species (pine and oak) and two different preservation states with a total of 80 wood samples were used to compare the results of different conservation methods with regards to their volume stabilisation. Wood is a very heterogeneous material, and the successful volume stabilisation of WAW depends on many factors^19,22,41,83^. This test series not only showed the great difference between the conservation methods investigated, but also the influence of the state of preservation and the type of wood on the result of certain conservation methods. Overall, all conservation methods contributed to a more or less good stabilisation of the samples compared to the air-dried, unconserved reference samples. The success of the volume stabilisation seems to be mainly related to the drying method. The methods with solvent-drying (alcohol-ether resin) and freeze-drying (PEG1 and PEG2) are the only ones with consistent good results concerning the volume stabilisation. It was found that the alcohol-ether method in particular stabilised the samples of both test series best, that there was no deformation along the wood anatomical directions and that neither cracks nor collapse in the wood structure were observed in the µCT data. The disadvantage of the method for practical application lies in the high safety requirements and the associated costs^50^. Thus, the method will probably remain limited to special objects, whereby the mode of action of stabilizing the cell walls^5,20^ is associated with the positive effect that the wood structure of such objects is clearly visible for non-destructive analyses^17,85^. This is not the case to the same extent with PEG methods with freeze-drying, as their mode of action is based on filling the pores to a greater or lesser extent^86^. In addition to the good results of volume stabilisation known from previous studies, the occurrence of fine cracks in the wood structure was also confirmed for PEG1 and PEG2^33,34^, which can be attributed to the necessary freezing prior to freeze-drying. The use of low molecular weight PEG in the PEG2 method reduces cracking, but further research is needed to improve this destabilizing deficiency for WAW objects of the most important practical methods. Kauramin 800 was unable to confirm the good results for volume stabilisation from the previous study^33,34^. The damage pattern in the form of large cracks along the annual rings in oak samples with two different states of preservation was also shown again. This seems to be due to stable chemical bonds with the lignin of the wood structure, which is also the reason for the legitimate criticism of the non-existent reversibility of the method^81,82^. Among the simple and cost-effective methods with air-drying used here (lactitol/trehalose, PEG4 and saccharose), Kauramin 800 still performs best. All other methods do not provide reliable results and confirm the occurrence of strong shrinkage and considerable collapse in the wood structure in one or the other wood species. However, there is a need for a method that allows the conservation of WAW for large objects and in the case of low resources. Also, the criticized mode of action through strong bonds with the wood components makes it worthwhile to continue researching melamine formaldehydes such as Kauramin 800, as they have the advantage that the wood structure is preserved for further wood-anatomic analyses and also remains clearly visible in the µCT. This is important, for example, for the conservation of dendrochronological samples.

However, these observations also show that further investigations and validation of the results are still necessary. µCT offers a method for reliably assessing the conservation-related changes in the wood. µCT measurements show the considerable damage such objects can take during conservation due to cracks and cell collapse. In addition, the complementary use of MRI also allows the water-saturated state before conservation to be recorded. The methodological approach presented here for the first time opens up new perspectives for the assessment of complex damage phenomena in WAW and allows better research into the causes of these damage patterns.

## Data Availability

The datasets generated and/or analysed during the current study are available on the repository Zenodo (https://doi.org/10.5281/zenodo.16535166, https://doi.org/10.5281/zenodo.16535029, https://doi.org/10.5281/zenodo.16534764). Further datasets used and/or analysed during the current study are available from the corresponding author on reasonable request.
